# Successful Management of Relapsing Primary Focal Segmental Glomerulosclerosis With Tacrolimus and Mycophenolate Multitarget Therapy

**DOI:** 10.1155/crin/4805412

**Published:** 2025-11-24

**Authors:** Frank Lee, Albert Yeam, Marrey Ruby Quizon, Wei Ling Lau

**Affiliations:** ^1^School of Medicine, University of California-Irvine, Irvine, California, USA; ^2^Division of Nephrology, Department of Medicine, University of California-Irvine, Irvine, California, USA

## Abstract

Focal segmental glomerulosclerosis (FSGS) is a leading cause of nephrotic syndrome, often resistant to corticosteroid therapy. We report a 75-year-old female with relapsing primary FSGS successfully treated with a dual regimen of tacrolimus and mycophenolate mofetil (MMF) without steroids or cyclophosphamide. Following three years of combination therapy, with concurrent renin–angiotensin system and sodium–glucose cotransporter-2 inhibition, the patient achieved sustained remission with proteinuria < 500 mg/day, without complications. This case underscores the potential of tacrolimus and MMF as a steroid-sparing strategy in managing refractory FSGS. Further research is needed to validate this approach in larger patient populations.

## 1. Background

Focal segmental glomerulosclerosis (FSGS) is the leading cause of end-stage renal disease among primary glomerular diseases in the United States and a common cause for nephrotic syndrome [1, 2]. FSGS describes a pattern of renal histological lesions demonstrating partial glomerular scarring, combined with podocyte injury and loss. Through a clinicopathological approach, FSGS is classified into primary, secondary, or genetic forms [3, 4].

Primary FSGS typically presents with an abrupt onset of severe nephrotic syndrome and is distinguished from other forms by electron microscopy, response to corticosteroids, and genetic screening [4]. It is hypothesized to be caused by a circulating extrarenal factor [5]. However, the exact pathophysiology remains unclear, and managing primary FSGS is challenging. First-line therapy involves corticosteroids, though relapses after remission are common. For steroid-resistant or intolerant patients, immunosuppressive therapies, including calcineurin inhibitors, mycophenolate mofetil (MMF), or biologics, have shown promise, although evidence is limited [6, 7].

Recent advances in multitarget immunosuppressive therapy have demonstrated potential in treating lupus nephritis and idiopathic membranous nephropathy [8–10]. In a multicenter randomized controlled trial, a multitarget therapy combining tacrolimus, MMF, and steroids achieved a higher incidence of complete remission and overall response in lupus nephritis patients, compared to cyclophosphamide and steroids [9]. Another study reported fewer adverse effects with a combination therapy of prednisone, cyclosporine, and MMF, compared to corticosteroid and cyclophosphamide treatment of idiopathic membranous nephropathy [10]. However, the feasibility of dual tacrolimus and MMF therapy for FSGS is unknown.

We present a case of relapsing primary FSGS successfully treated with a multitarget therapy of tacrolimus and MMF with years of stable disease remission. This case was previously presented as a poster at ASN Kidney Week 2024 [11], and here, we provide an expanded discussion with additional details and analysis.

## 2. Case Report

A 75-year-old female presented with recurrent nephrotic syndrome flares over several years. She had undergone two kidney biopsies, both of which revealed FSGS, not otherwise specified variant. The biopsies demonstrated diffuse podocyte foot process effacement, 30% glomeruli with segmental sclerosis, and 35% interstitial fibrosis. Her medical history was significant for chronic kidney disease Stage 3b, hypertension, well-controlled Type 2 diabetes mellitus, and dyslipidemia.

**Figure 1:**
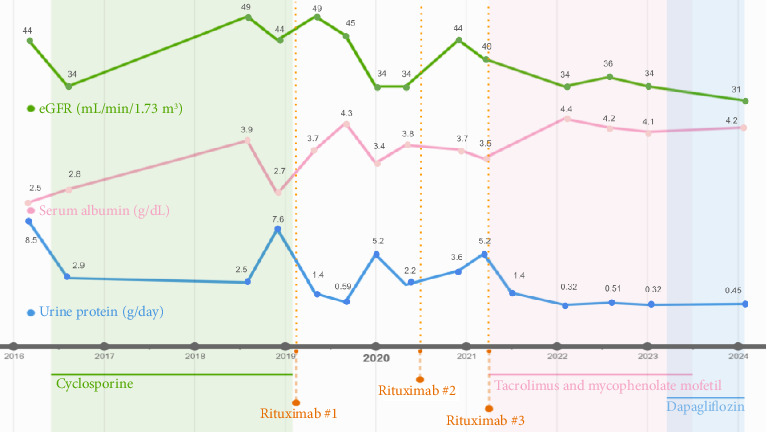
Trend of estimated glomerular filtration rate (eGFR), serum albumin, and proteinuria over 8 years.

At the time of initial diagnosis in May 2016, the patient exhibited severe proteinuria (8.5 g/day) on a stable dose of an angiotensin-converting enzyme inhibitor, benazepril 80 mg daily (Figure 1). The initial serum albumin was 2.5 g/dL with eGFR 44 mL/min/1.73 m^2^ (Figure 1). Treatment with first-line therapy, cyclosporine 75 mg BID (with stable blood trough levels of 100–175 ng/mL), resulted in partial remission, reducing proteinuria to 3.5 g/day (Figure 1). The patient declined steroid therapy due to concerns regarding potential side effects.

After 2 years on cyclosporine, the patient experienced a proteinuria flare of 7 g/day in 2018. Her treatment was changed to rituximab 375 mg/m^2^ weekly 4 doses. She responded well and proteinuria decreased to < 1 g/day with normalization of serum albumin to > 4 g/dL (Figure 1). However, she subsequently had annual FSGS flares with nephrotic syndrome, necessitating repeat rituximab treatments (total of three courses over 3 years).

In early 2021, the patient started oral multitarget therapy comprising low-dose tacrolimus 1 mg BID and MMF 500 mg BID. Blood tacrolimus trough levels ranged from 3.4 to 5.0 ng/mL. The tacrolimus dose was decreased to 0.5 mg BID in Nov 2021 due to hyperglycemia, and subsequent blood tacrolimus trough levels were 1.6–3.1 ng/mL. A sodium–glucose cotransporter-2 (SGLT2) inhibitor dapagliflozin 10 mg oral daily was added to her treatment regimen in early 2023. Following three years on this combination therapy, proteinuria remained stable (∼500 mg/day) without complications. Tacrolimus and MMF were weaned off by late 2023 (Figure 1).

## 3. Discussion

FSGS remains a leading cause of nephrotic syndrome in adults, with approximately 30%–50% of cases refractory to steroid treatment, underscoring the need for alternate therapies [12]. To date, we are the first to report successful treatment of recurrent FSGS with a steroid-free combination of tacrolimus and MMF. The patient achieved a prolonged, multiyear remission. Other FSGS cases treated with a combination of tacrolimus and MMF have included steroids or cyclophosphamide as part of their treatment protocol [13, 14].

Tacrolimus, a calcineurin inhibitor, acts by suppressing T-cell activation and has been shown to stabilize podocyte function and reduce proteinuria in resistant FSGS [15]. MMF inhibits lymphocyte proliferation by blocking inosine monophosphate dehydrogenase and is considered less nephrotoxic than calcineurin inhibitors when used alone [16]. Both agents have been evaluated recently for steroid-resistant or relapsing FSGS, often in combination with low-dose steroids [17]. Reported adverse effects of tacrolimus include nephrotoxicity, hypertension, neurotoxicity, and new-onset diabetes, while MMF is primarily associated with gastrointestinal symptoms, leukopenia, and increased infection risk [18, 19]. In current clinical practice, tacrolimus is typically administered at 0.05–0.1 mg/kg/day in two divided doses with target trough levels between 5 and 10 ng/mL, while MMF is dosed at 1–2 g/day [20, 21]. Treatment duration is variable but commonly ranges from 6 to 12 months, with dose tapering based on response and tolerability. The long-term steroid-free remission in our case supports further investigation of this multitarget regimen as a potential therapeutic option in primary FSGS while avoiding the significant side effect profile of steroids (hyperglycemia, weight gain, fluid retention, hypertension, bone demineralization, heightened risk of infection, and gastrointestinal ulcers).

The addition of an SGLT2 inhibitor also likely contributed to the patient's stable condition. SGLT2 inhibitors have been recognized for their renal-protective effects, particularly in reducing proteinuria and preserving kidney function [22]. However, their role in FSGS treatment is still under investigation [23].

This case underscores the importance of individualized treatment approaches for patients with primary FSGS, particularly for those resistant to conventional therapies. The combination of tacrolimus and MMF, potentially augmented by adjunct SGLT2 inhibitor therapy, represents a promising avenue for achieving sustained remission in this challenging patient population. Further research is needed to validate the efficacy and safety of dual tacrolimus and MMF treatment in managing primary FSGS.
